# Desynchronosis: Types, Main Mechanisms, Role in the Pathogenesis of Epilepsy and Other Diseases: A Literature Review

**DOI:** 10.3390/life12081218

**Published:** 2022-08-11

**Authors:** Elena D. Bazhanova

**Affiliations:** 1 Laboratory of Comparative Biochemistry of Cell Function, Sechenov Institute of Evolutionary Physiology and Biochemistry, Russian Academy of Sciences, 194223 St. Petersburg, Russia; bazhanovae@mail.ru; Tel.: +7-9119008134; 2 Laboratory of Morphology and Electron Microscopy, Golikov Research Center of Toxicology, 192019 St. Petersburg, Russia; 3 Laboratory of Apoptosis Studying, Astrakhan State University, 414040 Astrakhan, Russia

**Keywords:** circadian rhythms, gene regulation, clock, Bmal1, melatonin, desynchronosis, epilepsy, diseases

## Abstract

Circadian information is stored in mammalian tissues by an autonomous network of transcriptional feedback loops that have evolved to optimally regulate tissue-specific functions. Currently, stable circadian rhythms of the expression of clock genes (*Bmal1/Per2/Cry1*, etc.), hormones, and metabolic genes (*Glut4/leptin*, etc.) have been demonstrated. Desynchronoses are disorders of the body’s biorhythms, where the direction and degree of shift of various indicators of the oscillatory process are disturbed. Desynchronosis can be caused by natural conditions or man-made causes. The disruption of circadian rhythms is a risk factor for the appearance of physiological and behavioral disorders and the development of diseases, including epilepsy, and metabolic and oncological diseases. Evidence suggests that seizure activity in the epilepsy phenotype is associated with circadian dysfunction. Interactions between epilepsy and circadian rhythms may be mediated through melatonin, sleep–wake cycles, and clock genes. The correction of circadian dysfunction can lead to a decrease in seizure activity and vice versa. Currently, attempts are being made to pharmacologically correct desynchronosis and related psycho-emotional disorders, as well as combined somatic pathology. On the other hand, the normalization of the light regimen, the regulation of sleep–wake times, and phototherapy as additions to standard treatment can speed up the recovery of patients with various diseases.

## 1. “Biological Time”, Circadian Rhythms, Desynchronosis

A fundamental aspect of human physiology is the natural 24-h cycle, a feature inherent in life on Earth. The processes occurring in the body correlate with time, which contributes to survival, reflects the rotation of the planet, and corresponds to the daily rhythms of light and dark [1]. Natural cycles on Earth have different periodicities: these are daily, annual, and tidal cycles [2].

**Figure 1:**
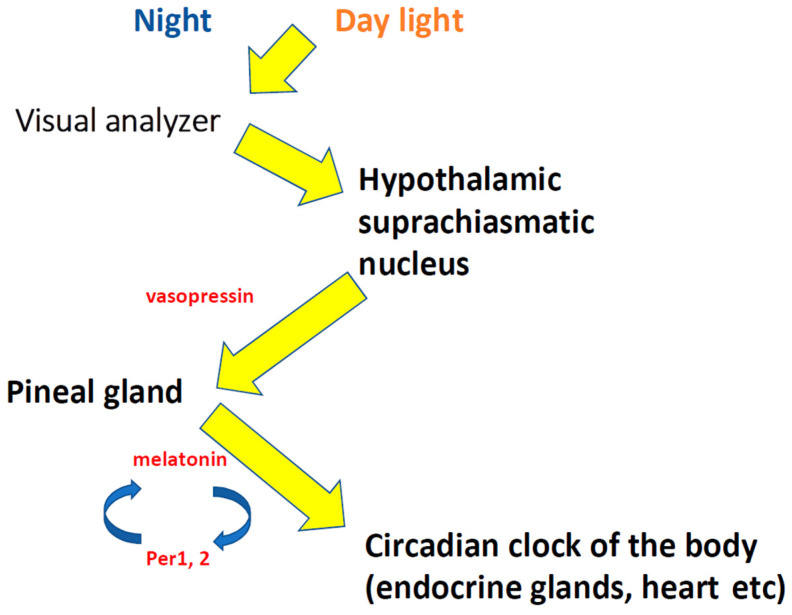
Regulation of the circadian clock (scheme).

Circadian rhythms are known to be cyclic fluctuations in the intensity of biological processes associated with the change in day and night, and their period is usually close to 24 h. Circadian rhythms are endogenous in origin, thus representing the body’s biological clock, although these rhythms are associated with external stimuli. The main organs that regulate biological time (the body’s work depending on circadian rhythms) are the visual analyzer [3], the pineal gland, and the suprachiasmatic nucleus (SCN) of the hypothalamus [4] (Figure 1). The suprachiasmatic nucleus (SCN) is the master circadian pacemaker in mammals and is entrained by environmental light [5]. Suprachiasmatic nuclei are small, paired nuclei adjacent to the optic chiasm [6]. The SCN consists of heterogeneous small neurons producing a wide range of neurotransmitters and neuromodulators [7]. Information from the photoreceptors of the retina through the fibers of the retinohypothalamic tract enters the SCN [8]. In response to stimulation, SCN cells produce neurohormone vasopressin. Vasopressin is one of the core signals via which the biological clock, the SCN, imposes its rhythm on its target structures, and its production and release is influenced by the rhythm of the clock genes and the light/dark cycle [9]. The parvocellular vasopressinergic neurons of the SCN synchronize the activity of the pacemaker in this nucleus. The SCN innervates the pineal gland to stimulate melatonin secretion at night. The pineal gland is also innervated by vasopressin- and oxytocin-containing fibers reaching the gland via a “central innervation” in the pineal stalk that may be involved in the annual regulation of melatonin secretion [10]. Throughout the body, such molecular clocks convey temporal control to the function of organs and tissues by regulating pertinent downstream programs. Synchrony between the different circadian oscillators and resonance with the solar day is largely enabled by a neural pacemaker, which is directly responsive to certain environmental cues and able to transmit internal time-of-day representations to the entire body [11]. The annual cycle of day length synchronizes annual rhythms, and, in mammals, this is mediated by nocturnal melatonin secretion proportional to night length [12]. The pineal hormone melatonin, an endogenous indolamine, is produced in the dark from tryptophan and plays a key role in the regulation of circadian rhythms generated by the SCN [4,13,14]. Melatonin performs many functions, including chronobiotic, antioxidant, and neuroprotective, has an antiinflammatory effect, and reduces damage caused by oxidative stress [15]. Melatonin is released into the blood and into the cerebrospinal fluid [4]. The melatonin molecule passes through the blood–brain barrier (BBB) and is able to enter into various subcellular components, such as the mitochondria and endoplasmic reticulum [16].

In addition to melatonin, glucocorticoids are also involved in the regulation of circadian rhythms and modulation of the protective response. The hypothalamic–pituitary–adrenal axis (HPAA) is the main neuroendocrine axis that regulates mammalian homeostasis. The glucocorticoid hormones are rapidly synthesized and secreted by the adrenal glands in response to stress. In addition, the basal level of glucocorticoids is formed rhythmically in response to circadian and ultradian (less than a day) patterns. These rhythms are important not only for the normal function of the target organs of glucocorticoids but also for the HPAA responses to stress. The disruption of glucocorticoid rhythms is associated with the development of diseases in both rodents and humans [17]. The interaction between the adrenal glands and the pineal gland under conditions of inflammation indicates that corticosterone potentiates nocturnal melatonin synthesis by reducing the activity of NFκB, a transcription factor that modulates the expression of key melatonin synthesis enzymes. It has been shown that the expressions of 70 out of 84 genes involved in the protective, adaptive responses of the body are significantly reduced at nightfall [18], which confirms the close relationship between stress hormones and circadian systems.

Desynchronoses are disorders of the body’s biorhythms, which consist of damage to the direction and degree of shift of various indicators of the oscillatory process. With desynchronosis, there is a change in the duration of the period, frequency, and amplitude of one or other biorhythms, and a mismatch in the previously synchronized intra- or intersystem rhythms. When the rhythms of the body are inconsistent with the rhythms of the external environment, external desynchronization is formed; when the rhythmic processes inside the body are inconsistent (at the level of the organs that form the functional system), then internal desynchronization develops.

There are various types of desynchronosis due to both natural conditions (natural lighting regimes in the north, in the Antarctic) and man-made causes (night shift work) (chronic desynchronosis). Desynchronosis can be caused by constant lighting, constant darkness, temporary disturbances of darkness during night sleep (even short-term flashes of light lead to circadian rhythm disturbances), etc. [19,20]. This type of disturbance also includes the so-called jet lag, classified as acute desynchronosis—damage to circadian rhythms caused by a rapid change in time zones (flight over long distances).

## 2. Molecular Genetic Regulation of Circadian Rhythms and Its Damage in Desynchronosis

Melatonin, released into the blood by the pineal gland at night in the absence of light, acts as a “hormone of darkness” on the brain and the body as a whole. Melatonin may also regulate the circadian rhythms of the SCN. During the change in day and night, melatonin exposure increases the activity of the central nervous system (CNS) through type 2 melatonin receptors, reducing the activation of protein kinase C. The effect of melatonin on the phasing of the CNS activity does not depend on daytime changes in the expression of the main genes involved in the molecular regulation of the circadian clock. The mammalian molecular circadian clock consists of a transcription–translation feedback loop composed of CLOCK (circadian locomotor output cycles kaput)-BMAL1 (aryl hydrocarbon receptor nuclear translocator-like) transcriptional activators and CRY (cryptochrome circadian regulator)-PER (period circadian regulator) transcriptional repressors [21]. Melatonin has been found to induce the expression of two clock genes, *Per1* and *Per2*, and this effect is mediated by protein kinase C. It can be assumed that the *Per1* and *Per2* genes are critical for resetting the photophase of the non-light biological clock (melatonin) (Figure 1). Regulation of these gene transcripts is necessary for the clock resetting caused by various regulatory signals [22].

Currently, stable circadian rhythms of clock genes’ expression have been demonstrated (*Bmal1* [3]/*Per2/Cry1/Cry2/NR1D1 (RevErbα*, nuclear receptor subfamily 1 group D member 1)) and for metabolic genes (*FAS* (fatty acid synthase)/*LPL* (lipoprotein lipase)/*Glut4* (facilitated glucose transporter, member 4)/*HSL* (hormone-sensitive lipase)/*leptin/visfatin/resistin*, etc.) (Table 1) [23]. Despite the dependence of metabolic gene expression on circadian rhythms, the distribution of fat depots is not associated with differences in the expression of the clock rhythm [23]. Changes in the expression of these genes under pathological conditions (development of hepatocellular carcinoma, administration of diethylnitrosamine, etc.) can be prevented by the administration of melatonin [24]. Melatonin significantly reduces proliferation and potentiates cell arrest. Melatonin treatment affects the expression of genes associated with apoptosis, inducing *p21, p53*, and *PARP1/2* expression and increasing the *Bax/Bcl-2* ratio. A decrease in *Bmal1* expression significantly reduces the proapoptotic and antiproliferative effects of melatonin [24].

Circadian information is stored in mammalian tissues by an autonomous network of transcriptional feedback loops that have evolved to optimally regulate tissue-specific functions. The analysis of daily gene expression in different tissues, as well as the assessment of intertissue circadian variability, are crucial for a systematic understanding of this transcriptional scheme. The authors used DNA microarray technology (Affymetrix) to study liver, muscle, adipose, and lung tissues obtained from animals after a series of circadian rhythm experiments. The analysis showed a high degree of circadian regulation with variable phase distribution in the four tissues studied. Interestingly, only a small number of common genes maintain circadian activity in all tissues, and many of them contain “core-clock” components with synchronous rhythms. These results indicate that intertissue circadian variability is a critical component of homeostatic body function and is mediated by various signaling pathways that ultimately lead to highly tissue-specific transcriptional regulation [25].

As is known, the circadian rhythms of behavior and physiology are coordinated by the endogenous clock located in the SCN of the hypothalamus. This central pacemaker also transmits day length information, providing seasonal adaptation that requires melatonin synthesis [26]. However, it is still unclear how the SCN encodes the length of the day. It has now been shown that adaptation to a short photoperiod is associated with the structural plasticity of the SCN independent of melatonin signaling; thus, the structural and, furthermore, the functional plasticity of the SCN play a key role in encoding day length. The miR-132/212 cluster (microRNAs, miRNAs are small non-coding RNAs that regulate gene expression by directing targeted miRNAs for degradation or translational repression) plays a key role in neuronal plasticity, and miR-132 has previously been shown to modulate the reset of central hours. It has recently been shown that miR-132/212 in mice is required for the regulation of the target gene, methyl-CpG-binding protein (*MeCP2*), in the SCN and the dendritic density of SCN neurons. The result is optimal adaptation to the seasons and the 24-h light/dark cycle [26].

Other authors also consider microRNA miR-132 to be a key regulator of plasticity-related processes in the CNS and one of the regulators of the circadian system. All this, taken together with the found miR-132 expression in the hippocampus, where it affects neuronal morphology and memory, prompted testing of the idea that the circadian rhythms of this miRNA in the forebrain modulate cognitive functions depending on diurnal fluctuations. Experiments on miR-132-knockout and transgenic mice revealed the effect of miR-132 on MeCP2 and Sirt1, two miR-132 targets that are associated with neuronal plasticity and cognition. In mice of both studied lines, the rhythmic expression of MeCP2 and Sirt1 was suppressed. Thus, it is highly likely that miR-132 is the key by which the circadian system communicates the daily rhythm to cognition [27].

**Table 1:** Some genes involved in regulation of circadian rhythms.

Name	Official Full Name	Function
*CLOCK (KAT13D)*	*c* *ircadian locomotor output cycles kaput*	The protein encoded by this gene plays a central role in the regulation of circadian rhythms. The CLOCK protein encodes a transcription factor of the basic helix–loop–helix (bHLH) family and has a DNA-binding histone acetyltransferase activity. The CLOCK protein forms a heterodimer with ARNTL (BMAL1), which binds the E-box enhancer elements upstream of the *period* (*PER1*, *PER2*, *PER3*) and *cryptochrome* (*CRY1*, *CRY2*) genes and activates the transcription of these genes. Polymorphisms of this gene may be associated with behavioral changes in certain populations, as well as with obesity and metabolic syndrome [23,28].
*ARNTL (BMAL1)*	*aryl hydrocarbon receptor nuclear translocator-like*	The protein encoded by this gene forms a heterodimer with CLOCK. This heterodimer binds E-box enhancer elements upstream of the *period* (*PER1*, *PER2*, *PER3*) and *cryptochrome* (*CRY1*, *CRY2*) genes and activates the transcription of these genes. The PER and CRY proteins heterodimerize and repress their own transcription by interacting in a feedback loop with the CLOCK/ARNTL complexes. Defects in the *BMAL1* gene are associated with infertility, problems with gluconeogenesis and lipogenesis, and altered sleep patterns. The protein regulates gene expression stimulated by interferon and is an important factor in viral infection, including COVID-19 [3,24,28].
*PER1*	*period circadian regulator 1*	This gene is a member of the period gene family and is expressed in a circadian pattern in the SCN. The genes of this family encode components of the circadian rhythms of motor activity, metabolism, and behavior. The *PER1* gene is activated by CLOCK/ARNTL heterodimers, but then represses this activation in a feedback loop using PER/CRY heterodimers to interact with CLOCK/ARNTL. *PER1* polymorphisms may increase the risk of certain types of cancer [22,23,28].
*PER2*	*period circadian regulator* *2*	This gene is a member of the period gene family and is expressed in a circadian pattern in the SCN. The genes of this family encode components of the circadian rhythms of motor activity, metabolism, and behavior. *PER2* is activated by CLOCK/ARNTL heterodimers, but then represses this activation in a feedback loop using PER/CRY heterodimers to interact with CLOCK/ARNTL. *PER2* polymorphisms may increase the risk of certain types of cancer and are associated with sleep disorders [22,23,28].
*CRY1*	*cryptochrome circadian regulator 1*	*CRY1* encodes a protein that binds flavin adenine dinucleotide, which is a key component of the circadian master oscillator complex that regulates the circadian clock. This gene is activated by CLOCK/ARNTL heterodimers, but then represses this activation in a feedback loop using PER/CRY heterodimers to interact with CLOCK/ARNTL. Polymorphisms in *CRY1* are associated with changes in sleep patterns. The CRY1 protein is conserved in plants and animals. Loss of a related gene in mice shortens the circadian cycle in complete darkness [23,28].
*CRY2*	*cryptochrome circadian regulator 2*	This gene encodes a flavin adenine dinucleotide-binding protein that is a key component of the circadian core oscillator complex, which regulates the circadian clock. This gene is upregulated by CLOCK/ARNTL heterodimers, but then represses this upregulation in a feedback loop using PER/CRY heterodimers to interact with CLOCK/ARNTL. Polymorphisms in *CRY2* have been associated with altered sleep patterns. The CRY2 protein is conserved across plants and animals [23,28].
*NR1D1 (RevErba)*	*nuclear receptor subfamily 1 group D member 1*	This gene encodes a transcription factor that is a member of the nuclear receptor subfamily 1. The encoded protein is a transcription factor that negatively regulates the expression of major clock proteins. Specifically, this protein represses nuclear translocator protein 1, circadian clock transcription factor, and aryl hydrocarbon receptor protein 1 (ARNTL). This protein may also be involved in the regulation of genes involved in metabolic, inflammatory, and cardiovascular processes [23,28].

## 3. Changes in Biological Parameters in Desynchronosis: The Role of Desynchronosis in the Etiopathogenesis of Diseases

Life on Earth has evolved over the past few billion years under conditions of relatively bright days and dark nights. The widespread use of electric light during the past century, and the consequent significant light at night, are important factors affecting both humans and animals for the first time in evolutionary history. The endogenous circadian clock depends on light in the external environment, and seasonal rhythms depend on clear nocturnal melatonin signals, which are different at various times of the year. Thus, light at night can disrupt melatonin synthesis and thus temporal adaptations [29,30]. Indeed, disruption of the light–dark cycles that naturally appeared in evolution leads to quite serious changes in physiology, behavior, and mood [29], i.e., significantly affects the functions of the nervous system.

It has now been shown that the morphology of neurons and the neuronal network are very plastic and change during the life of animals, including humans, in response to various stimuli. The number of synapses and the shape of neurons change during the day, oscillate in accordance with circadian rhythms generated by the endogenous biological clock, or depend directly on stimuli coming from the external environment. Such changes are called circadian plasticity, and they are extremely important for sensory processing, learning, and memory. The disruption of circadian rhythms can cause significant damage to brain function [31].

The so-called “light pollution” damage to photoperiodism leads to the development of desynchronosis and, further, to premature aging and the emergence of various pathological changes, including age-related diseases [20] and oncological diseases [30,32], and the deterioration in existing diseases, such as epilepsy [33,34]. Changes in the light–dark regime lead to the disruption of various body systems, primarily reproductive [35].

A study of the so-called “Antarctic Syndrome”, which occurs when staying at a station in the Antarctic for 1 month or more, showed the appearance of multiple symptoms of dysregulation and maladaptation as a result of the functional stress of the sympathetic–adrenal system. Studies have shown a significant increase in the level of catecholamines (adrenaline, norepinephrine, dopamine, DOPA) in the urine and markers of oxidative stress in the blood plasma, the activation of free radical processes, a decrease in the activity of SOD (superoxide dismutase, an antiradical enzyme), an increase in delta rhythms, and other changes in electrical activity brain, characteristic of hypoxia [19].

Experimental data indicate that desynchronosis induced by the constant lighting or natural light regimes of the northern regions, knockout or mutation of the clock genes, pinealectomy, and jet lag leads to dysfunction of the pineal gland, which in turn stimulates the development of tumors, various transplanted, spontaneous and induced carcinogens, in laboratory animal models [36].

### 3.1. The Risk of Metabolic Diseases

The disruption of circadian rhythms is a risk factor for the development of physiological and behavioral disorders, including weight gain and metabolic diseases. Thus, night shift workers are predisposed to obesity and metabolic dysregulation, which is the result of the disruption of circadian rhythms. Although human studies rely on correlation, studies using experimental models have identified several mechanisms by which light at night can excite these metabolic effects by disrupting inflammation-associated circadian rhythms both in the brain and in the periphery. Eating disruption is a key effect of light at night and subsequent metabolic dysregulation [29].

Light exposure at night disrupts the circadian system in adult animals, leading to arrhythmia in nocturnal rodents. In the early stages of development, the circadian system has a critical period of adjustment, at which time it is particularly vulnerable to changing lighting conditions that can program its function with long-term consequences. It was shown that the loss of rhythm in this case is associated with a decrease in the number of VIP (vasointestinal peptide)-, vasopressin-, and PER1-immunopositive cells in the SCN, which indicates the risk of damage to the light–dark cycle in early ontogenesis [37].

Chronic sleep deprivation is an important social problem associated with an increased risk of metabolic diseases. Mouse models show that damage to circadian rhythms affects the pathogenesis of obesity [38]. Hypothalamic inflammation leads to hyperphagia and weight gain (dietary obesity), but sleep deprivation-induced neuroinflammation can also lead to metabolic dysfunction. Experiments on C57BL/6J mice have shown that sleep disturbance causes an inflammatory response in brain regions that regulate energy balance and exercise glycemic control. A comparison of the concentrations of proinflammatory cytokines in central and peripheral metabolic tissues indicated that the patterns of interleukin-1β concentrations reflect the observed changes in glucose tolerance. In addition, mice with disturbed sleep showed the activation of microglia (increased expression of Iba1) [39]. However, some authors deny the influence of circadian rhythms on the distribution and deposition of adipose tissue in the body [23].

Melatonin has been shown to influence insulin secretion, and this action is mediated by melatonin receptors MT1 and MT2. In vivo and in vitro experiments have confirmed that insulin is secreted in the pancreas in accordance with circadian rhythms, due to the action of melatonin on MT1 and MT2, which induce cell function. Melatonin induces the production of IGF (insulin-like growth factor) and promotes the phosphorylation of insulin receptors. Thus, melatonin may be involved in the pathogenesis of diabetes, as evidenced by a decrease in the level of this hormone and a decrease in the functional relationship between melatonin and insulin in diabetic patients. It has been shown that the imbalance of the internal circadian systems induces glucose intolerance and insulin resistance, which can be restored by the administration of melatonin. In addition, it has been shown that the presence of melatonin receptors on human pancreatic islets may influence the pharmacotherapy of type 2 diabetes [14].

### 3.2. Changes in the Cardiovascular System

In humans, circadian rhythms have been extensively studied in the cardiovascular system. Many studies have shown the importance of the circadian clock in all major cell types and organs of the cardiovascular system. Daily rhythms play an important role in the physiology and diseases of the cardiovascular system. Endogenous, approximately 24-h circadian rhythms in the brain, autonomic nervous system, heart, and vascular system prepare the cardiovascular system for optimal functioning during behavioral cycles [40,41,42,43,44]. Many cardiovascular functions such as endothelial function, thrombus formation, blood pressure, and heart rate are regulated by the circadian clock. The peripheral clocks in the smooth muscle, perivascular adipose tissue, liver, adrenal gland, and kidney have been recently implicated in the regulation of blood pressure rhythm. Dysregulation of the circadian rhythm of blood pressure is associated with adverse cardiorenal outcomes and an increased risk of cardiovascular mortality [44]. The circadian rhythm also manifests itself in pathology—at the onset of acute myocardial infarction, stroke, arrhythmia, and other adverse cardiovascular events. Cardiovascular circadian rhythms can be dangerous. Normal heightened responses in the morning can facilitate the transition from sleep to activity, but such exaggerated responses are potentially harmful to people prone to adverse cardiovascular events. Indeed, the occurrence of stroke, myocardial infarction, and sudden cardiac death have daily patterns, most often in the morning [41,43].

This suggests that circadian rhythms may serve a therapeutic purpose by using or altering molecular time to improve existing treatments and develop new ones [43]. It is necessary to emphasize the importance of the circadian system for normal cardiovascular function and cardiovascular diseases, as well as to identify opportunities for optimizing the timing of drug administration in cardiovascular diseases [41]. Clinically meaningful effects of melatonin treatment have been demonstrated in placebo-controlled trials in humans, particularly in disorders associated with diminished or misaligned melatonin rhythms, for example, circadian rhythm-related sleep disorders, jet lag and shift work, insomnia in children with neurodevelopmental disorders, poor (non-restorative) sleep quality, non-dipping nocturnal blood pressure (nocturnal hypertension), and Alzheimer’s disease [42]. Dysfunctions of the cardiovascular system, especially those associated with psycho-emotional stress, occur in healthy people as a result of disruption to the sleep–wake cycle due to shift work, and a classic example is the desynchronization of locomotive drivers. Similar conditions develop in connection with significant somatic pathology, for example, in cancer patients [45].

### 3.3. Epilepsy

The link between circadian rhythms and epilepsy has been established for decades. Epilepsy is highly circadian in nature. Interactions between epilepsy and circadian rhythms may be mediated through melatonin, sleep–wake cycles, and clock genes. However, many questions underlying the complex mechanisms of their interaction remain unresolved. The study of these interactions will allow the development of an accurate seizure detection algorithm and alternative precise therapeutic strategies. The role of circadian rhythms in the pathogenesis of epilepsy has been studied and characterized in human studies as well as in animal studies using various models of epilepsy. Preclinical laboratory studies based on animal models of epilepsy with controlled epileptogenic pathology and a range of intervention strategies have revealed bidirectional effects between the circadian rhythm and seizures, as well as the underlying mechanisms [33,34]. There is growing evidence that an intrinsic disruption of the circadian molecular system can lead to epileptogenesis. This is supported by genetic models of animals with the loss of circadian proteins (e.g., CLOCK33 and PAR bZIP15 proteins) that develop seizures in vivo [33]. However, there is other evidence that the initial epileptic seizure causes disruption to circadian rhythms. Numerous studies with pilocarpine and kainate models of epilepsy show that the initial acute epileptogenic seizure with the use of these drugs caused acute changes in the circadian molecular system (for example, an increase in Per1 and Reverbα, as well as a decrease in Rorα (RAR-related orphan receptor alpha)) [46,47]. There appears to be a bidirectional relationship between epilepsy and circadian dysfunction. Circadian dysfunction can lead to the formation of an epileptic network, and an epileptic seizure can lead to acute circadian dysfunction [33].

We consider epilepsy to be a disease associated with desynchronosis because, in patients, not only do physiological rhythms change, but new ones associated with the disease are formed [48]. Research shows that different types of epilepsy present with different circadian signatures, depending on the semiology of the seizures and the location of the lesion. Patients with epilepsy have disturbances in sleep patterns and the sleep–wake cycle, which are behavioral manifestations of the circadian rhythm. In patients with epilepsy, the circadian rhythm has also been shown to influence the autonomic response, hormonal rhythm, and response to anticonvulsants. Despite the relatively well-characterized circadian nature of epilepsy, our understanding of the circadian mechanisms regulating seizures is incomplete at the cellular and molecular level.

Various studies have shown that epilepsy occurs more frequently at certain points in the 24-h cycle. These data point to the overlap of circadian rhythms and epilepsy; however, the molecular and cellular mechanisms underlying this circadian regulation are still unclear. It is known that the 24-h rhythm is generated by the circadian molecular system, and, therefore, this system, consisting of many circadian genes, is involved in epilepsy. Various circadian genes such as *Clock, Bmal1, Per1, Rev-erbα,* and *Rorα* are involved in the regulation of epilepsy. In various animal models of epilepsy, the circadian fluctuations and steady state levels of these genes are disrupted. The descending pathway of these genes includes a large number of metabolic pathways associated with epilepsy. These pathways include the metabolism of pyridoxal, the mammalian target of the rapamycin pathway, and the regulation of redox balance. It is hypothesized that disruption of these metabolic pathways may mediate the circadian regulation of epilepsy. A better understanding of the cellular and molecular mechanism of circadian regulation in epilepsy will allow precise targeting of circadian rhythm disturbances in epilepsy for a novel therapeutic approach [33].

There have been numerous studies showing that circadian rhythm disturbance is associated with certain types of seizures. In experimental epilepsy, partial seizures fold into a regular circadian pattern, indicating that the person’s seizures also meet the precise criteria for circadian rhythms [48].

Seizures occur in different circadian patterns depending on the location of the seizure onset zone. Seizures originating in the frontal lobes occur predominantly during sleep, while temporal and occipital seizures predominate during wakefulness. In addition, the circadian rhythms of seizure occurrence also depend on the types of seizures. Tonic, tonic–clonic, and hypermotor seizures are more likely to occur during sleep, while clonic, myoclonic, and hypomotor seizures are more likely to occur during the daytime [49].

Despite several new antiepileptic drugs, nearly 30–40% of patients with epilepsy continue to have seizures. However, among patients with drug-resistant epilepsy, only a small proportion are suitable for surgical treatment of epilepsy. There is a medical need to provide better control of seizures in these patients. Possible treatments for epilepsy in the future are chronotherapy and neural stem cell transplantation. Chronotherapy is a therapeutic intervention aimed at improving the efficacy and tolerability of drugs by determining the optimal timing of drug administration from a circadian perspective. Numerous studies have shown promising results in optimizing the frequency of seizures in patients with epilepsy. Once the temporal pattern of seizures is recognized, chronotherapy can be performed by administering a conventional medication at the appropriate time. In addition, since there is a strong association between clock genes and epilepsy, chronotherapy can be achieved by applying external cues that reset circadian rhythms [49]. In most animal models, a study of anticonvulsant treatment has shown an improvement in circadian dysfunction, which generally correlates with seizure reduction and severity [50].

Thus, research data suggest that seizure activity in the epilepsy phenotype is associated with circadian dysfunction. The correction of circadian dysfunction may lead to an improvement in seizure activity and vice versa.

### 3.4. Social Jet Lag

However, it is not only shift work, but also sleep disturbances associated with the use of electronic media (computers, smartphones, etc.) that can lead to desynchronosis, causing a state of “social jet lag”. Most often, this pathology is observed in adolescents because sleep is an important factor in the development of the body. Shortening of sleep time (falling asleep late and waking up early due to school activities) leads to the desynchronization of rhythms and loss of sleep. In addition, the use of electronic devices before bedtime is a factor of concern since LEDs emit much more blue light than white incandescent and compact fluorescent lamps and therefore have a greater impact on the biological clock. The result of all this is an imbalance of biological and social rhythms, a lack of sleep leading to daytime sleepiness, behavioral problems, and academic failure [51].

### 3.5. Desynchronosis Caused by Damage to the Light–Dark Ratio

The absence of a change in light and darkness (constant lighting or constant darkness) affects the physiological parameters that depend on the circadian rhythms of the body (the activity of the antioxidant system, the immune system). However, changes in parameters not associated with circadian fluctuation (e.g., vitamin concentration, puberty, aging) depend on the level of melatonin produced by the pineal gland [52].

Using a model of Wistar rats, it was shown that desynchronosis caused by constant illumination affects the quantitative and qualitative parameters of bone marrow cells, while the introduction of melatonin normalizes this effect [53]; with this type of desynchronosis, the number of melatonin-positive cells of the gastric mucosa of rats is significantly reduced, in almost all age groups [54].

The effect of illumination on biomarkers of oxidative stress in a model of hyperlipidemic nephropathy induced by adriamycin administration was revealed as a possible result of the relationship between the secretory rhythms of melatonin and leptin. The introduction of adriamycin stimulates oxidative stress in the kidneys, brain, liver, and heart. In healthy animals, and to a lesser extent in nephropathy, constant light increases oxidative stress, while darkness improves the condition. This is probably due to the release of melatonin in the dark phase and the secretion of leptin in the light phase. The correlation between melatonin and leptin levels in healthy animals confirms the relationship between these parameters and their impact on oxidative stress [55].

Experimental modeling of the jet lag effect on laboratory animals (guinea pigs) showed an increase in the levels of anxiety and depressive behavior, the deterioration in long-term memory and cognitive abilities, and spatial disorientation. The level of melatonin in the peripheral blood decreased, while the concentration of cortisol increased. The study of the immune status revealed a decrease in the concentration of interleukin-4 and interferon-gamma in blood plasma. Thus, changes in behavior and cognitive abilities are obviously associated with damage to the immune status caused by desynchronosis [56].

Other authors also confirm the active participation of the immune system in the pathogenesis of desynchronosis. Thus, the effect of constant illumination on the state of the central and peripheral organs of the immune system of rats of different genetic lines (ISIAH and WAG) was revealed. In ISIAH rats, constant illumination for 2 weeks resulted in the inhibition of cell proliferation and differentiation in the thymus, a marked decrease in splenocyte proliferation, and a decrease in the number of T and B cells in the spleen compared with control WAG rats, although the level of antigen-presenting cells in the spleen of ISIAH rats rose. The authors concluded that the ISIAH rats were more sensitive to changes in illumination than the controls. Thus, 24-h illumination leads to damage to the central differentiation of T cells and the activation of systemic inflammation associated with the dysregulation of cell differentiation, which enhances the metabolic dysfunction in these animals [57]. In experiments with chronic constant lighting on mouse models of different genetic lines (CBA, C57BL/6), genotype-dependent reactions of the nervous and immune systems were also revealed [58].

In addition, internal desynchronosis can be induced not only by a change in the light regime but also by other causes, for example, chemical pollution of the environment [59].

## 4. Pharmacological Correction of Desynchronosis and Changes in the Body Caused by Synchronism: Phototherapy for Various Diseases

The term “chronopathology” reflects the loss of harmony of circadian rhythms and the emergence of risk factors that are critical for patients. In modern medicine, the concept of “chronofitness” has been introduced as a new target for the treatment of various diseases, pursuing the goal of internal synchronization of the biological clocks of various tissues, as well as external synchronization with the environment. A chronobiological approach to the treatment of various pathological conditions is proposed, including a multicomponent strategy that promotes chronofitness, and further research in the development of personalized medicine associated with the normalization of biological rhythms [1].

The studies of many authors have shown the etiopathogenetic role of desynchronosis in the development of various diseases. Thus, experimental and epidemiological studies show that the destruction of the circadian clock plays an important role in epilepsy, carcinogenesis, and the development of metabolic diseases. Cellular clocks outside the brain are efficiently coordinated by the rhythm of body temperature. It is assumed that simultaneous measurements of body temperature and resting activity rhythms contribute to the assessment of individual circadian clock coordination in patients, which allows the integration of biological rhythms into precision medicine. It has been shown that the determination of the circadian rhythm of body temperature is clinically important, both for assessing the impact on the health of people with atypical work schedules and for identifying key determinants of circadian disruption in cancer patients [60].

At present, attempts are being made to correct desynchronoses and related psycho-emotional disorders, as well as combined somatic pathology. Thus, the drug Prolit Super Septo, as part of a combination therapy for patients with infectious and inflammatory diseases of the urinary system, reduces inflammation, improves clinical symptoms, corrects psycho-emotional status, and restores normal chronorhythms, regardless of the location of inflammation [61]. The positive effect of the complex drug “NeuroDoz” in the treatment of patients with erectile dysfunction was revealed, and the psycho-emotional status and chronorhythms were also normalized [62].

It has been shown that melatonin can reduce headaches caused by various causes, especially in cluster headaches and migraines [13]. However, there are studies that deny the involvement of extra-epiphyseal melatonin and dietary melatonin in the regulation of the biological clock function in vertebrates [4].

Alzheimer’s disease (AD) is a neurodegenerative disease characterized by β-amyloid deposition in the extracellular matrix and tau proteins in neurons. Many factors play a role in the development of AD, such as familial mutations, oxidative stress, and post-translational changes. It is assumed that melatonin, as an antioxidant and neuroprotector, can play a positive role in AD therapy by regulating enzymes involved in peroxidation and protecting mitochondria from hyperoxygenation [16]. It has also been shown that Parkinson’s disease is characterized by non-motor symptoms such as sleep and circadian rhythm disturbances. The melanopsin photopigment and retinal photoreceptors involved in the regulation of body biological rhythms are already damaged at an early stage in patients with Parkinson’s disease [63]. Thus, genes that regulate circadian rhythms can serve as targets for pharmacological correction in these neurodegenerative diseases.

In experiments, it was found that sympathectomy, light deprivation, hibernation, and the introduction of melatonin have an inhibitory effect on carcinogenesis [36].

On the other hand, the normalization of the light regime, the regulation of sleep–wake time, and the use of phototherapy as an addition to standard treatment can significantly accelerate the recovery of patients with various diseases, including, for example, chronic bacterial cystitis [64]. The sleep–wake patterns of patients during hospitalization provide significant opportunities for improving hospital care [65]. Circadian rhythm disorders (sleep disorders, mood disorders) caused by lack of light and orexin imbalance respond well to bright light therapy during the day [66].

## 5. Conclusions

Thus, it is obvious that circadian rhythms determine the functioning of the whole organism and each of its systems, and damage to these rhythms, both acute and chronic (desynchronosis), leads to significant damage to the functions of various systems and organs. In our review, we showed that desynchronosis of any genesis contributes to the appearance and progression of the pathology of various body systems, and the emergence and worsening of diseases that were not previously associated with circadian rhythms (for example, epilepsy, etc.). On the other hand, various diseases can intensify desynchronosis, creating a so-called vicious circle (for example, mental pathologies, such as depression, etc.).

However, there are still many unresolved questions. Thus, dysregulation of the circadian pattern of blood pressure, with or without hypertension, is associated with an increased risk of cardiovascular disease. The mechanism of this dysregulation is unknown and is a growing area of research. More tissue-specific studies of the mechanism of the molecular clock are needed, as well as clinical studies involving more diverse populations (different races, women, etc.), which will be critical for a complete understanding of the mechanism of the circadian regulation of blood pressure. A better understanding of peripheral clock function in regulating the circadian rhythm of blood pressure will help pave the way for targeted therapeutics in the treatment of circadian blood pressure dysregulation and hypertension [40,44]. The same can be noted for other diseases, and one of the most important is epilepsy, in which approximately 40% of patients suffer from a drug-resistant form. Growing evidence shows the bidirectional interactions between sleep, circadian rhythm, and epilepsy. Comprehending how these interact with each other may help to advance our understanding of the pathophysiology of epilepsy and develop new treatment strategies to improve seizure control by reducing the medication side effects and the risks associated with seizures. Further research is needed to elucidate the pathways by which rhythmic patterns of epileptic activity are generated as this may also influence future treatment options.
